# Real-World Use of Topical Ruxolitinib in Vitiligo: Retrospective Cross-Sectional Mixed Methods Infodemiology Study of the r/Vitiligo Subreddit

**DOI:** 10.2196/78247

**Published:** 2025-10-21

**Authors:** Michael Constantin Kirchberger, Carola Berking, Andreas Eisenried

**Affiliations:** 1 Dermatology Center Ingolstadt, Schlüterstr. 3a, Ingolstadt, 85057, Germany, 49 0841 4936936; 2 Department of Dermatology, Uniklinikum Erlangen, Deutsches Zentrum Immuntherapie, Friedrich-Alexander-Universität Erlangen-Nürnberg, Erlangen, Germany; 3 Lehrstuhl für Anästhesiologie Friedrich-Alexander-Universität Erlangen-Nürnberg, Erlangen, Germany

**Keywords:** vitiligo, ruxolitinib, Reddit, infodemiology, digital epidemiology

## Abstract

**Background:**

Vitiligo significantly impairs quality of life. Topical ruxolitinib is a novel Janus kinase inhibitor approved for nonsegmental vitiligo, but real-world patient experiences, particularly regarding efficacy, side effects, and access challenges following approval, are not fully captured by clinical trials. Online patient forums like Reddit offer valuable insights into these aspects.

**Objective:**

This study analyzed discussions on the r/Vitiligo subreddit regarding topical ruxolitinib to understand real-world patient experiences, perceived treatment success, side effects, access barriers, and overall sentiment.

**Methods:**

We conducted a retrospective, cross-sectional infodemiology study of posts and comments mentioning ruxolitinib or Opzelura on r/Vitiligo between January 2022 and December 2024. After filtering and preprocessing, 2950 entries were analyzed. Computational linguistics (all-MiniLM-L6-v2), including sentence-transformer embeddings for semisupervised topic classification into therapy success, side effects, insurance and cost, and off-topic, were used. Valence Aware Dictionary and Sentiment Reasoner (−1 to +1) for sentiment analysis was assessed. Temporal trends were analyzed; model performance was validated manually against blinded manual annotation of 500 entries. Representative qualitative data were reviewed.

**Results:**

Discussions increased following regulatory approvals. Therapy success was the largest cluster (entries: 1765/2950 , 59.83%; 95% CI 58.1 to 61.6) with positive sentiment (mean score 0.473, 95% CI 0.46 to 0.48), frequently describing facial repigmentation and adjunctive use with phototherapy. Users reported encouraging hair repigmentation within treated areas and success even on vitiligo spots present for over 20 years, while noting that areas like hands and feet were particularly treatment-resistant. The side effects cluster (entries: 558/2950, 18.91%; 95% CI 17.5 to 20.3) had negative sentiment (mean score −0.110, 95% CI −0.14 to 0.07), frequently mentioning application-site acne, fatigue, and panic attacks or anemia. The insurance and cost cluster (entries: 491/2950, 16.64%; 95% CI 15.3 to 18.0) had positive sentiment (mean score 0.349, 95% CI 0.31 to 0.39), dominated by discussions on high costs and access difficulties, alongside strategies like co-pay programs but also noting insurance denials. Manual model validation showed substantial agreement (accuracy 88.4%, 95% CI 86 to 91; *F*_1_-score 0.893, 95% CI 0.865 to 0.918; Cohen κ 0.801, 95% CI 0.760 to 0.840).

**Conclusions:**

Real-world Reddit narratives broadly corroborate clinical trial efficacy signals, particularly facial repigmentation and utility alongside phototherapy, while highlighting practical barriers including frequent application-site acne and cost or insurance friction. These findings have direct clinical and policy implications: clinicians can proactively counsel about the expected benefit on facial areas, monitor and manage acne or irritation, and discuss combination with phototherapy; practices and payers can mitigate access delays using before authorization templates and co-pay assistance. Social media infodemiology thus complements pharmacovigilance and health services research by quantifying patient-reported outcomes and surfacing access issues at scale, informing patient counseling and coverage decisions in routine care.

## Introduction

Vitiligo is a chronic autoimmune skin disorder characterized by patchy loss of pigmentation, affecting approximately 0.5% to 2% of the global population [1]. Beyond its physical manifestations, the condition often leads to significant psychosocial distress, including depression, anxiety, metabolic syndrome, and stigmatization, impairing patients’ quality of life [2-4].

British guidelines from 2022 advocate a stepwise approach to vitiligo management. For limited disease, potent anti-inflammatory creams (topical corticosteroids or calcineurin inhibitors) are recommended, escalating to light therapy using a specific wavelength of UV light (narrowband UV-B phototherapy) or systemic corticosteroids in progressive or extensive cases [5]. More recent European guidelines corroborate this and acknowledge the emergence of novel therapies, such as topical Janus kinase inhibitors (JAK) like ruxolitinib, as promising options for patients with nonsegmental vitiligo [6,7].

The phase 3 TRuE-V1 (Topical Ruxolitinib Evaluation in Vitiligo Study) and TRuE-V2 trials demonstrated its efficacy in nonsegmental vitiligo [8]. At week 24, 29.8% and 30.9% of patients achieved ≥75% improvement in the Facial Vitiligo Area Scoring Index, a clinical tool for measuring repigmentation on the face. The treatment was well-tolerated, with the most common adverse events being acne and pruritus at the application site.

Based on these positive efficacy and safety data, ruxolitinib cream (Opzelura) was approved by the US Food and Drug Administration (FDA) in July 2022 for the treatment of nonsegmental vitiligo in individuals aged ≥12 years [9]. The European Commission followed in April 2023, authorizing its use for the same indication with facial involvement [10]. Subsequent approvals were granted by the UK Medicines and Healthcare Products Regulatory Agency in July 2023 and by Health Canada in October 2024. Collectively, these regulatory decisions reflect the growing recognition of topical JAK inhibitors as a valuable therapeutic option in the management of nonsegmental vitiligo.

However, regulatory approval alone does not guarantee patient access to new therapies. National health technology assessments and reimbursement decisions often create disparities, as exemplified by the initial National Institute for Health and Care Excellence recommendations against ruxolitinib cream in the United Kingdom and similar concerns in Canada regarding its long-term cost-effectiveness and quality-of-life impact compared to existing treatments.

This disconnect between regulatory approval and reimbursement can restrict access, leading patients to seek information and support on online platforms, particularly social media, which offer insights into lived experiences often missed in clinical settings.

This patient-driven shift to online platforms has given rise to the scientific field of infodemiology, defined as the study of the distribution and determinants of information on the internet to inform public health [11]. A core application is infoveillance, the process of using this data for real-time public health surveillance. Reddit, in particular, has emerged as a valuable ecological data source for infodemiology studies due to its topic-specific communities (subreddits) that foster candid discussions. For instance, Reddit data were used to understand the emotional journey of patients and caregivers navigating brain cancer diagnoses [12]. Furthermore, this approach has proven effective in analyzing the broader information landscape, such as successfully characterizing the prevalence of misinformation and stigma in discussions about obesity [13]. Among the various social media platforms, Reddit has emerged as a particularly valuable data source for such research.

Reddit, ranked as the 7th most visited website in the United States, offers pseudonymous, topic-specific forums such as r/Vitiligo, capturing authentic patient perspectives on treatment efficacy, emotional impacts, and barriers to health care access. Advances in natural language processing and machine learning now enable systematic analyses of Reddit’s user-generated content, transforming authentic patient conversations into valuable real-world data. The primary aim of this study was to systematically analyze patient-generated discussions on the r/Vitiligo subreddit to characterize the real-world experiences with topical ruxolitinib. To achieve this, we used a retrospective, cross-sectional analysis using computational linguistics and semisupervised machine learning. Specifically, we sought to (1) categorize discussions into key thematic clusters, focusing on perceived efficacy, side effects, and issues of cost and access; (2) analyze the sentiment within each theme to understand patient attitudes; (3) track discussion volume over time in relation to key regulatory approvals, ultimately uncovering overarching patient experiences and treatment attitudes; and (4) qualitatively explore representative patient narratives to add context and depth to the quantitative findings.

## Methods

### Study Design

To understand the real-world patient journey with topical ruxolitinib, this study analyzed thousands of authentic, unsolicited conversations from the r/Vitiligo online community. Our methodology follows a retrospective, cross-sectional mixed-methods framework, integrating large-scale computational analysis with qualitative human interpretation to provide a robust and nuanced picture. The process began by collecting a comprehensive dataset of relevant posts and comments. We then used a 2-pronged computational linguistics pipeline. First, a semisupervised machine learning model, guided by clinically relevant examples, automatically classified each discussion into one of three core themes: therapy success, side effects, or insurance and cost. Second, the Valence Aware Dictionary and Sentiment Reasoner (VADER) algorithm, a tool tailored for social media text developed by Hutto and Gilbert [14], assessed the emotional sentiment of each post. To ensure the reliability of our automated approach, the classification model’s performance was rigorously validated against a manually annotated sample. Finally, to contextualize the quantitative data, we conducted an exploratory qualitative review, examining representative patient narratives to uncover the specific experiences behind the trends.

### Data Source and Filtering

All posts and comments from the subreddit between January 2022 and December 2024 were collected. The dataset was then specifically filtered to retain entries explicitly mentioning ruxolitinib or its commercial name Opzelura. The deleted or empty posts and comments were removed. Standard text preprocessing techniques were applied, including converting text to lowercase, deleting special characters, and lemmatization using the WordNet algorithm (Princeton University).

### Temporal and Seasonal Analysis

Monthly posting and commenting frequencies were aggregated, and a 12-month moving average was calculated to smooth short-term variations and reveal long-term trends. Seasonal activity patterns were examined by calculating average monthly activity across each month over the entire study period.

### Semisupervised Topic Classification

We adopted a semisupervised machine learning approach using sentence-transformer embeddings (all-MiniLM-L6-v2 by *sentence-transformers*) to categorize posts into predefined thematic clusters. Three clinically relevant clusters were established: therapy success, side effects, and insurance and *c*ost*,* each represented by 7 domain-specific prototype phrases.

Document embeddings, representing each post or comment as a vector, were computed and then compared with the averaged embeddings of the predefined prototype phrases using cosine similarity.

A keyword-based override mechanism supplemented the semantic classification, allowing reassignment of documents if specific lexical indicators strongly suggested alternative classifications.

### Sentiment Analysis

Sentiment of postings was analyzed using VADER. VADER assesses the sentiment of textual data, assigning a continuous score ranging from strongly negative (−1.0) to strongly positive.

### Validation Procedure

To evaluate the reliability and accuracy of our classification model, we conducted a structured validation involving a human rater. A random proportional sample of 500 entries across all categories was independently evaluated presenting the post or comment, and classification according to the 4 clusters had to be chosen. The performance was quantified using standard classification metrics like accuracy and the *F*_1_-score (a combined measure of the model’s precision and recall), while interrater agreement was measured by Cohen κ coefficient (a statistic that shows how closely the model’s classifications matched a human’s, accounting for the possibility of agreement by chance). Based on established benchmarks, κ values are interpreted as follows: 0.01‐0.20 as slight, 0.21‐0.40 as fair, 0.41‐0.60 as moderate, 0.61‐0.80 as substantial, and 0.81‐1.00 as almost perfect agreement.

### Software and Tools

Data analysis was performed using Python (version 3.12.7; Python Software Foundation) and the following libraries: *pandas* (version 2.2) for data handling, *sentence-transformers* (version 2.7) for embeddings, *vaderSentiment* (version 3.3) for sentiment analysis, *ruptures* (version 1.1.10) for change-point detection, and *Matplotlib* (version 3.8) for visualization.

### Exploratory Qualitative Review of Content

For illustrative purposes, we screened representative posts within each major cluster to extract noteworthy patient experiences and recurring concerns, aiming to highlight insights potentially relevant for improving patient care.

### Ethical Considerations

This study was conducted using exclusively publicly available and anonymized data from the r/Vitiligo subreddit. No personally identifiable information that could lead to the identification of individuals was collected or analyzed. The research adhered to established ethical standards for studies involving publicly accessible online data, ensuring that user privacy and data confidentiality were preserved throughout all stages of data collection and analysis. In accordance with guidelines, a formal ethics committee review and individual informed consent were not required.

## Results

### Data Cohort and Filtering

**Figure 1.**
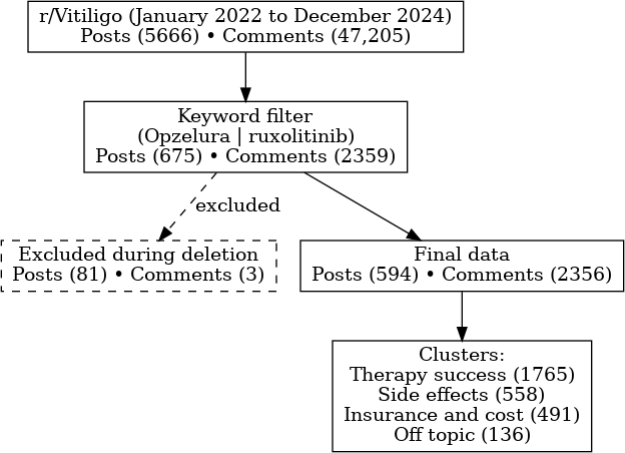
Study flow diagram. Sequential filtering of 52,871 reddit entries to the final analytic cohort of 2950 ruxolitinib-related posts or comments.

A total of 52,871 user-generated entries (5666 posts and 47,205 comments) were collected from the r/Vitiligo subreddit between January 2022 and December 2024. Application of keyword filtering for ruxolitinib or Opzelura yielded 3034 relevant entries (675 posts and 2359 comments). After removing 81 posts and 3 comments due to empty content, the final dataset comprised 2950 unique entries (594 posts and 2356 comments) for analysis (Figure 1).

### Temporal and Seasonal Patterns

**Figure 2.**
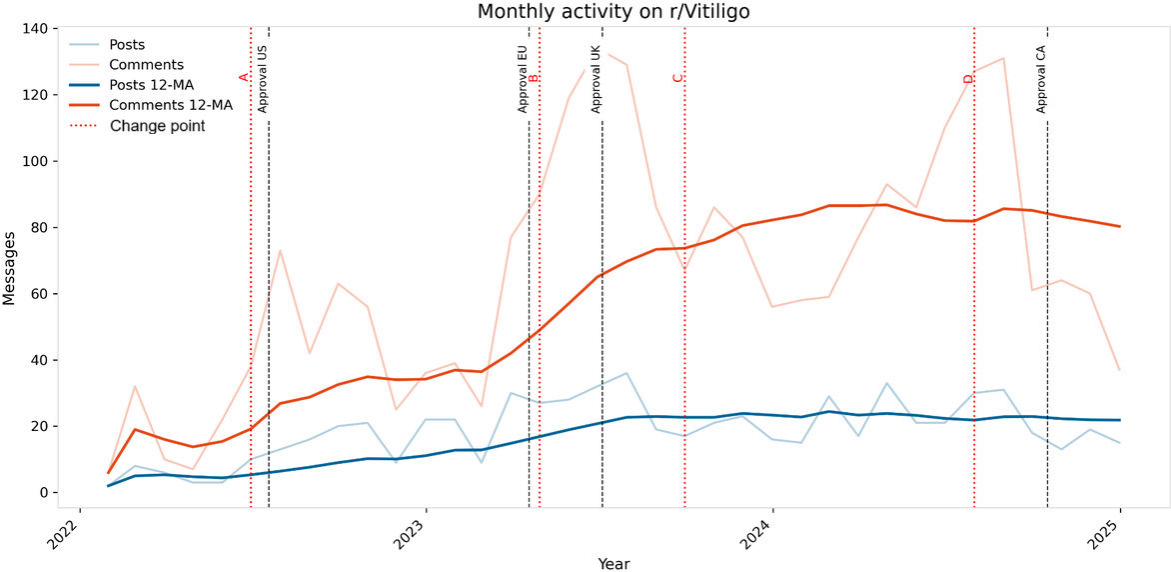
Monthly discussion volume (2022‐2024). Lines show posts and comments per month; vertical dashed lines mark US Food and Drug Administration (FDA; July 2022), European Medicines Agency (EMA; April 2023), and UK Medicines and Healthcare Products Regulatory Agency (MHRA; July 2023), and Health Canada (October 2024) approvals of ruxolitinib cream. MA: moving average.

Analysis of monthly posting and commenting frequencies revealed an upward trend in discussion volume over the study period (Figure 2). The 12-month moving average for comments, in particular, showed a notable increase, especially following regulatory approvals in the United States (July 2022), European Union (April 2023), United Kingdom (July 2023), and Health Canada (October 2024). Automatic change-point analysis identified significant shifts in discussion volume corresponding to these approval milestones.

**Figure 3.**
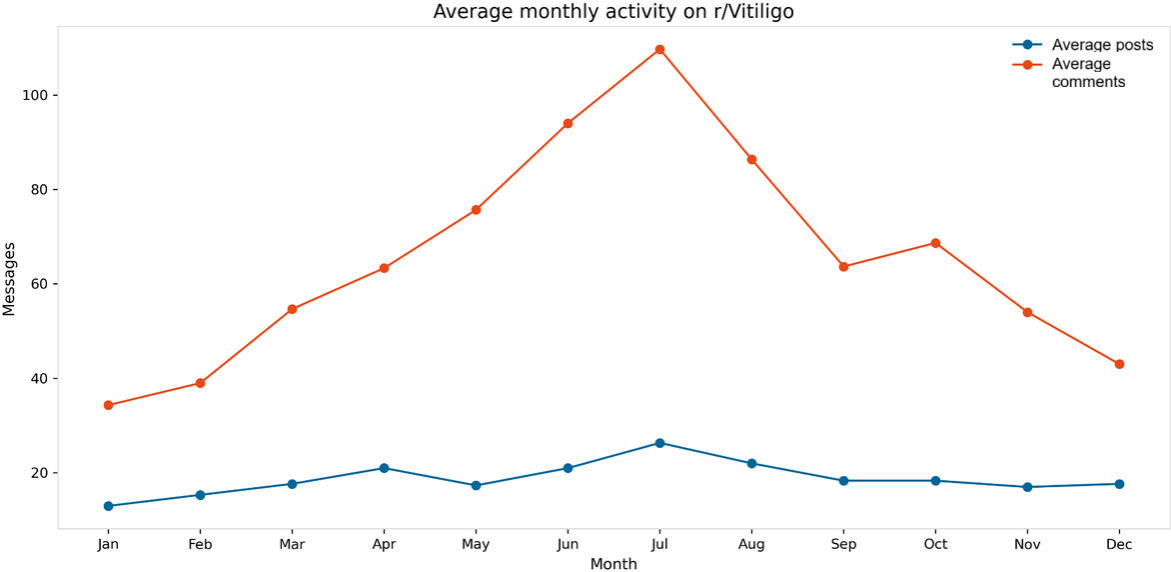
Seasonal activity pattern. Mean number of posts and comments aggregated by calendar month; highest engagement occurs in July.

Examination of seasonal activity patterns indicated peak discussion levels during the summer months, with July exhibiting the highest average number of both posts and comments (Figure 3). Comment activity consistently exceeded post activity across most months. Specifically, comment activity was greater than post activity in 9 out of 12 months, with a median monthly difference of +57 (IQR 19‐88) messages. This difference was statistically significant in 2-tailed tests (Wilcoxon signed-rank test: W=15, *P*=.01; paired 2-tailed *t* test: *t*_11_=3.17, *P*=.01; Cohen *d*_z_=0.67).

### Sentiment Analysis

**Figure 4.**
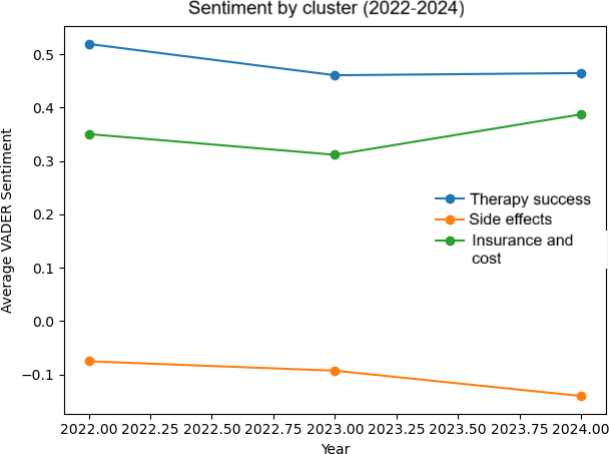
Valence Aware Dictionary and Sentiment Reasoner (VADER) sentiment across thematic clusters.

Sentiment analysis using VADER revealed distinct emotional tones across the thematic clusters (Table 1 and Figure 4). Discussions within the therapy success cluster exhibited a notably positive average sentiment score of 0.473 (95% CI 0.46 to 0.48), indicating a consistently optimistic and supportive tone in these discussions. The insurance and cost cluster also showed a positive sentiment of 0.349 (95% CI 0.31 to 0.39), though less pronounced. In contrast, the side effects cluster demonstrated a negative average sentiment score of −0.110 (95% CI −0.14 to 0.07), reflecting a clear tone of frustration, concern, and dissatisfaction. The off-topic cluster had a mildly positive sentiment of 0.236 (95% CI 0.20 to 0.27)*.*

**Table 1.** Distribution and mean Valence Aware Dictionary and Sentiment Reasoner (VADER) sentiment of thematic clusters from the r/Vitiligo subreddit.

Cluster name	Items, n (%; 95% CI)	Average VADER sentiment score, mean (95% CI)
Therapy success	1765 (59.83; 58.1 to 61.6)	0.473 (0.46 to 0.48)
Side effects	558 (18.91; 17.5 to 20.3)	−0.110 (−0.14 to 0.07)
Insurance and cost	491 (16.64; 15.3 to 18.0)	0.349 (0.31 to 0.39)
Off-topic	136 (4.61; 3.9 to 5.4)	0.236 (0.20 to 0.27)

Over the study period (2022‐2024), sentiment within the therapy success cluster remained consistently positive, showing a slight downward trend. Sentiment in the side effects cluster was consistently negative and declined slightly over time. In contrast, sentiment related to insurance and cost was generally positive and improved modestly throughout the observed period (Figure 4).

### Validation of Classification Model

The performance of the semisupervised topic classification model was evaluated through manual annotation of a proportional random sample of 500 entries. The model demonstrated 88.4% (95% CI 86 to 91) accuracy and a weighted *F*_1_-score of 0.893 (95% CI 0.865 to 0.918). Interrater reliability, as measured by Cohen κ coefficient, was calculated at 0.801 (95% CI 0.760 to 0.840). Taken together, these strong metrics confirm that our automated analysis was highly reliable and sorted patient discussions with a level of accuracy that closely mirrors human judgment.

### Qualitative Results

#### Therapy and Success

Many users described significant improvements, especially for facial vitiligo:

*Face cleard 90% in some 4 months.*

One user stated:

*I have been using ruxolitinib with UV-B and I have seen drastic improvements after only 2 months now. My first spot underneath my right eye has almost completely closed up.*

Another user shared a long-term perspective:

*Then last year (20+ y after the first diagnosis) it started spreading again. So I went to the dermatologist and learned about all the new treatments and started Opzelura about 3 months ago and it seems to be working pretty well. Even the original spots that are over 20 years old are starting to fade!*

The potential for hair repigmentation was also noted:

*In just a little over 2 months on opzelura I’ve had hair repigment from white/clear to black and my spot drastically close in.*

*I have repigmented eyebrow hair eyelash hair and beard hair with Opzelura.*

Efficacy on long-standing vitiligo was also highlighted:

*I’ve cured areas with 30+ years of vitiligo.*

The face was consistently reported as the area most responsive to Opzelura:

*Opzelura is probably the best cream you can get for your face.*

*Face repigments the fastest because you have the most hair follicles there.*

Eyelids were also mentioned as an area of successful treatment:

*My eyelids are almost fully repigmented*

*I was able to repigment my eyelids very quickly using Opzelura and UV laser therapy.*

Combination therapy, particularly with UV-B light, was frequently mentioned as beneficial:

*Yes UV-B is basically the golden standard when it comes to repigmenting and stabilizing vitiligo. When using UV-B in combination with JAK inhibitors or elidel/tacrolimus you will get the best results.*

Similarly, success was found with the Excimer laser:

*Yes I was able to repigment all areas that had a hair follicle pretty quickly with Opzelura+excimer.*

Some users found Opzelura superior to previous treatments:

*From my experience elidel didn’t do anything except for stop the spread of spots.*

*In 80 g so far of opzelura plus some sunlight, I’ve already seen repigmentation faster than the tacrolimus NB-UB-B (narrowband UV-B) combo.*

An interesting observation was the repigmentation of white hair in treated areas:

*Also have seen a large amount of black hair throughout my eyebrow and beard.*

*Opzelura turned a lot of my beard hair jet black.*

However, this was not universal:

*My dermatologist actually has told me even prior to starting opzelura that my skin tone has completely repigmented BUT my beard still has big white patches. So the hair color hasn’t returned yet.*

Another user stated:

*Been using Opzelura for 6 months, applying on areas with white hair. Seen no results*.

#### Failures and Limitations

However, not all experiences were positive. Some users reported limited success or failures:

*I’ve been using opzelura for a year and it hasn’t really helped much alone.*

Another user mentioned:

*Been on Opzelura for 6 months, applying on areas with white hair. Seen no results.*

The cream’s effectiveness appeared to vary by body region, with hands and feet being notably difficult to treat. One user commented:

*Opzelura seems to be working pretty well for most areas except hands and feet.*

This was echoed by another:

*Opzelura is known to not work well for hand and feet. They state that on their website.*

*Haven’t used it on my hands. Heard that success on hands was very limited, so don’t want to waste it on hands as they are almost completely depigmented, and pigmentation might just make them stand out.*

Someone else noted:

*I have been using opzelura on my hands and feet for about half a year and those spots have not changed much. If anything, opzelura has stabilized the spots on my hands and feet.*

Some users did not see results even after extended use:

*Been using opzelura for 9 months, oral steroids for 6 months and UV-B for 2 months, and I have had zero progress and continuous progression.*

*Keep it your luck to have it all the same color. opzelura hasn’t worked at all for me it’s been 8 months.”*

The cessation of treatment could lead to relapse:

*Once you stop opzelura it relapses, which is why they are pursuing the t-cell therapy that will address the relapse issue.*

*The results are mixed. The top pic is what I started with, the middle one is when I applied it every day and night for a good 7+ months. It seems to do well but it hits a plateau where it just won’t fill in more on those areas...The bottom pic is now. After not using it for another 5 months or so. It looks like they mostly came back to before pic.*

### Side Effects

#### Commonly Reported Side Effects

The most frequently mentioned side effect was acne at the application site:

*The only side effect I have experienced is acne but it’s just like a white head here and there and subsided within 2 days. You’ll likely only get that side effect if you’re treating the face.*

Some experienced skin irritation:

*I’ve found that I sometimes get itchy in the areas where I’ve applied Opzelura.” and “A couple days ago, I started experiencing burning, hive like bumps, and swelling on my neck.*

#### Fatigue

Fatigue was specifically mentioned by some users:

*Anyone using Opzelura notice any side effects? Asking because I am using it (twice a d) and notice some fatigue or malaise.*

Another user shared:

*I stopped using because I was running out of prescription but I also I’m experiencing fatigue during the day a lot more when I have it on.*

*I actually had horrible side effects from Opzelura even with a small dose, so I had to stop. I was super fatigued every day.*

Another one reported:

*Just a warning. I used opzelura for a year and 6 months. At some point started craving ice… now today I have severe anemia and it’s difficult to do anything at all due to extreme fatigue.*

#### Alcohol Interaction

One user mentioned:

*Apparently, some people experience an alcohol-induced flush wherever tacro has been applied—so keep that in mind if you drink and experience the flush. It almost always happened to me after having a beer or some wine. The skin would get [very] red that it was pretty embarrassing.*

Another user on Opzelura mentioned their alcohol consumption without noting an interaction:

*My vitiligo on my face is 90% gone after 9 months of opzelura. I drink a couple beers everyday after work, a little more than a couple on weekends.*

#### Other Side Effects and Concerns

Some users experienced other side effects like a metallic taste:

*Anyone have a metallic or funny taste in your mouth since using the cream?*

or increased illness:

*I’ve been using opzelura for a couple of months I have vitiligo under my armpits. I’ve noticed that I get sick more often now than I have in the past.*

One user reported panic attacks:

*I used opzelura for 3 weeks and started very soon to experience [panic] attacks.*

Concerns about the black box warning associated with JAK inhibitors (the class of drugs to which ruxolitinib belongs) were also present, though users often differentiated between the risks of oral versus topical application:

*A lot of dermatologists mentioned the black box warning being for oral ruxolitinib as it results in high concentrations of the drug in the blood, which causes the side effects. For the topics version, it was tested in 2 clinical trials for a year each time and the only reported side effect in like 15% of the patients was acne in the location.*

*The side effects are for oral JAK, not topical opzelura.*

However, one user shared a more severe experience:

*My doctor gave me Opzelura and it almost killed me. It didn’t take too long less than two weeks. It was the topical version. Yes, the topical version.*

### Insurance and Cost

#### Cost and Insurance Coverage

Opzelura is noted to be expensive. One user mentioned:

*Opzelura is very expensive cream and insurances may be resistive in covering it.*

The out-of-pocket cost can be substantial:

*I was told $2000 a tube, dermatologist connected me to the hospitals pharmacy and there was some $0 plan and they deliver it. I think it was this copay program they mentioned*

*In the US, Opzelura costs over $2000.*

Many users reported difficulties obtaining insurance approval:

*My insurance originally denied it, so I called them and asked whats up… they told me the dermatologist didn’t submit all of the paperwork.*

Another user shared:

*Upon hearing of the FDA approval—I got an appointment last week with my dermatologist; she immediately wrote me the Rx for Opzelura and sent it to my location pharmacy. I got a call back a day later that they would not fill it, and I need a pre-authorization. Got a call back 2 days later that the pre-authorization was denied by my insurance company.*

Some insurers deemed the treatment “cosmetic”:

*My insurance company makes me fail on a few treatments until Opzelura is approved...fepBlue BCBS does not recognize Opzelura in any capacity or in any pricing tier for the treatment of Vitiligo. They say it is “cosmetic.”*

#### Patient Assistance Programs and Co-Pay Cards

To mitigate costs, users mentioned patient assistance programs and co-pay cards:

*If you don’t qualify for assistance, then you can explore other options that are not the same thing but work well.*

The IncyteCARES program was frequently cited:

*If you are in the US, text “save” to 91,830 and they will send u an Opzelura Co-Pay card with $0 copay*

*My dermatologist referred me to Incyte for this medication as he wants me to try it. I’ve been dealing with Incyte directly for the last few days and its been nothing but going in circles so far.*

One user detailed their success:

*My insurance company denied covering Opzelura so my dermatologist had my Rx sent to NimbleRX where I bought it for $35 and it was delivered to my front door the following day.*

Another user reported:

*I got Opzelura for free!!! I live in the US and have […] insurance and was able to get it for free with Optum Specialty Pharmacy?*

#### Alternative Sourcing (Compounding or Overseas)

Some users explored compounding pharmacies or purchasing generic versions from other countries due to cost or lack of availability:

T*here are compounding pharmacies in the US that produce the cream from Ruxolitinib pills. Chemistry RX has been offering 1 g per $10, their product works well.*

However, the legality and quality of such sources were questioned:

*It’s not legal for any pharmacy to compound a patented drug unless it’s significantly different that the commercially available drug.*

*Ruxolitinib (the drug itself) indeed not. These are manufactured in India, without any approval/review of some FDA. You’re not totally 100% sure what’s in it, it problably will be some sort of copy cat.*

Experiences with ordering from overseas varied:

*I’ve used india mart to order the generic ruxolitinib cream sent to uk. I can’t say I’ve had much success with using the cream though.*

One user stated:

*I have been using ruxolitinib and tofacitinib creams ordered from India to the USA. I have placed multiple orders with this vendor and have not had any issues. The creams have worked as well.*

#### Selling or Trading Medication

There were instances of users offering to sell or trade Opzelura.

*I have opzelura for sale at fair prices. Also have referrals from costumers.*

*If anyone is interested in buying opzelura dm me I am done with treatment and have atleast 5 treatments left.*

*Anyone needing some Opzelura I have 2 unopened tubes I’m willing to part with as I’m in a Metformin clinical trial now and cannot continue with this treatment.*

Others posted about having extra tubes:

*I have extra Opzelura, message me.*

*I have a brand new tube of opzelura that I never got to use because my vitiligo went away. If anyone is interested in buying I’m willing to let go for much much cheaper then retail.*

### Off-Topic

Some users expressed how vitiligo affected their self-esteem and intimacy. One user shared:

*Mine started when I was in fourth grade and I am now 22. Super hard for me since it was the shaping years of my life... has led to OCD, anxiety, depression and a condition called vaginismus. It has been the root of intimacy issues.*

Another user described a negative comment from their husband:

*Unfortunately my worst commentator is my husband, seems by skin bothers him more than anyone, opzelura is working on my arms and hands, and now I really don’t care what he thinks about the rest of me.*

There were also accounts of supportive partners. One user recounted an experience with their now-husband:

*He looked up and said, “How cute, you have a spot under your chin.*

Another offered encouragement:

*Hang in there brother. Most women will accept it... your family, true friends and that special someone will love you just as you are.*

One user mentioned their ex-boyfriend’s perspective:

*My ex boyfriend said he dated me because of my vitiligo he said “I dated you because you looked unique, you looked different! I loved your spots because no one else I know has them” as we were breaking up…”*

### Health Care Provider Interactions

In addition, frustrations with health care providers surfaced, exemplified by comments such as:

*My Lux dermatologist refuses to prescribe opzelura due to side effects. Same with the GP.*

*Any dermatologist that diagnoses you with vitiligo and refuses to prescribe for a condition which opzelura is FDA approved to treat would be considered a moron unless you have a contraindication of some sort.*

*no worries. yeah your derm is just an [poor physician] if they were dismissive about your concerns.*

These exchanges illustrate the broader emotional, social, and practical contexts in which patients navigate their treatment journeys.

## Discussion

### Overview

By analyzing a substantial dataset of patient-generated discussions on the r/Vitiligo subreddit, this study offers a unique real-world perspective on the experiences with topical ruxolitinib. Our thematic analysis revealed that patient conversations were primarily dominated by discussions of therapy success (59.83%, 95% CI 58.1 to 61.6), followed by significant concerns regarding side effects (18.91%, 95% CI 17.5 to 20.3) and insurance and cost (16.64%, 95% CI 15.3 to 18.0). This thematic distribution was mirrored in our sentiment analysis, which uncovered a strongly positive attitude toward the treatment’s efficacy (mean score 0.473, 95% CI 0.46 to 0.48), contrasted by a negative perception of its side effects (−0.110, 95% CI −0.14 to 0.07*).* These patient perceptions evolved over time, as discussion volume surged in response to key regulatory approvals, while our qualitative review of individual narratives provided crucial context to these trends by illuminating the human experiences behind the data. Collectively, these findings provide a comprehensive, data-driven overview of the patient journey, highlighting a complex interplay of therapeutic optimism and practical challenges.

### Principal Findings

The most prominent theme in our analysis was therapy success, which garnered the highest volume of discussion and a consistently positive sentiment. This aligns with the established efficacy of ruxolitinib cream, particularly for facial vitiligo, as demonstrated in the pivotal TRuE-V1 and TRuE-V2 trials, where approximately 30% of patients achieved improvement in the Facial Vitiligo Area Scoring Index by week 24 [8]. Our qualitative data corroborated these findings, with numerous users reporting significant repigmentation, especially on the face, and often when ruxolitinib was used in conjunction with phototherapy, particularly UVB light. This synergy between topical JAK inhibitors and phototherapy is an area of active investigation, with real-world patient accounts suggesting a perceived enhanced benefit, which warrants further exploration in controlled settings. The observation of hair repigmentation within treated areas by some users is also noteworthy, reflecting a potential for follicular melanocyte activation that adds to the understanding of the drug’s mechanism and patient-valued outcomes.

The *side effects* cluster was characterized by a distinctly negative sentiment. Our qualitative analysis confirmed that the most frequently reported adverse event was application-site acne, which is consistent with the safety profile observed in clinical trials. Beyond trial data, real-world evidence from formal channels like the FDA Adverse Event Reporting System has identified signals for specific systemic events, including anemia and headache [15]. Our analysis of Reddit discussions, however, uncovered a different spectrum of patient-reported systemic effects, capturing more nuanced concerns such as fatigue, a metallic taste, and panic attacks. This highlights the unique value of infodemiology; while formal reporting systems effectively capture defined clinical outcomes, social media analysis is a powerful tool for discovering a broader range of idiosyncratic, quality-of-life-impacting side effects that patients experience and discuss in their own words. While the black box warning for oral JAK inhibitors casts a shadow, users often distinguished the perceived risks of topical versus systemic administration. Nevertheless, the reporting of significant adverse events, albeit anecdotally and by a small number of users (eg, one user claiming the topical version “almost killed me” and another linking it to severe anemia), underscores the value of pharmacovigilance through social media listening to identify potential safety signals that may not be apparent from trial data alone. The slight increase in negative sentiment within this cluster over time could reflect a growing cohort of users experiencing longer-term exposure or a broader patient population beginning treatment. For dermatologists, this highlights the importance of proactively counseling patients on potential side effects like application-site acne and systemic fatigue, thereby managing expectations and improving treatment adherence.

The *insurance and cost* cluster revealed the significant practical hurdles patients face in accessing ruxolitinib. Despite its regulatory approvals, numerous discussions centered on high out-of-pocket costs, difficulties obtaining insurance before authorization, and insurers deeming the treatment *cosmetic*. This directly mirrors the complex reimbursement landscape where bodies like the National Institute for Health and Care Excellence in the United Kingdom and the Canadian Drug Expert Committee have expressed concerns regarding cost-effectiveness and the translation of clinical repigmentation into patient-reported quality of life improvements, leading to restricted access through public health care systems. The relatively positive sentiment in this cluster, and its slight improvement over time, may seem counterintuitive but can be explained by users actively sharing strategies to overcome these barriers, such as using manufacturer co-pay programs (eg, IncyteCARES), navigating specialty pharmacies, or even resorting to purchasing compounded or generic versions from overseas, albeit with concerns about legality and quality. This finding underscores Reddit’s role as a platform for peer-to-peer support, echoing the conclusions of Rajanala et al [16], who found that patients with chronic conditions like inflammatory bowel disease use the platform for “emotional support” and “crowdsourcing information” to navigate gaps in traditional care.

The temporal analysis of discussion volume showed a clear correlation with major regulatory milestones, with notable increases in posts and comments following FDA, European Medicines Agency, and UK Medicines and Healthcare Products Regulatory Agency approvals. This suggests that regulatory news directly fuels patient interest and information-seeking behavior within these online communities. The observed seasonality, with peak activity during summer months, may be attributable to increased visibility of vitiligo lesions due to sun exposure and greater social interaction or simply more leisure time for online engagement. The consistently higher volume of comments compared to posts underscores the interactive nature of these forums, where initial posts often trigger extensive discussions.

The qualitative insights derived from patient narratives provide a rich, nuanced understanding that often goes beyond structured clinical trial data. For example, the commonly reported perception that hands and feet are particularly resistant to ruxolitinib, even acknowledged on the manufacturer’s website according to one user, reflects real-world treatment limitations.

Furthermore, reports of relapse upon treatment cessation highlight the ongoing need for maintenance strategies or therapies that can induce more durable remission, a concern that patient discussions bring to the forefront. The off-topic discussions, which touched upon the psychosocial impact of vitiligo on intimacy and self-esteem, and interactions with healthcare providers, reaffirm the profound burden of this condition and the importance of patient-centered care that addresses both the physical and emotional aspects of vitiligo.

### Limitations

This study has several limitations inherent to the methodology. First, Reddit users do not represent the entire patient population with vitiligo; they are likely to be younger, more technologically adept, and potentially more proactive in seeking information, introducing a selection bias. Also, users could predominantly be English-speaking and often come from higher-income countries. The anonymity of the platform, while fostering open discussion, means that reported experiences cannot be clinically verified, and diagnoses or treatment details are self-reported. Second, while computational linguistics and sentiment analysis tools like VADER are powerful, they are not infallible and can misinterpret sarcasm, nuanced language, or complex contexts, despite our validation procedure showing substantial agreement (Cohen κ 0.801, 95% CI 0.760 to 0.840). The semisupervised machine learning approach, while effective with an accuracy of 88.4% (95% CI 86 to 91) and a weighted *F*_1_-score of 0.893 (95% CI 0.865 to 0.918), is dependent on the predefined thematic clusters, potentially overlooking emergent themes not captured by these categories. Third, this is a retrospective observational study, so no causal inferences can be drawn. Finally, while r/Vitiligo is an international forum, the timing of discussions may be skewed by approvals and launches in specific geographies, notably the United States.

### Conclusions

This analysis of patient discussions on the r/Vitiligo subreddit offers valuable, real-world insights into the multifaceted experiences with topical ruxolitinib. While patient narratives corroborate trial data regarding efficacy, particularly for facial vitiligo, they also highlight significant challenges related to side effects (including those less emphasized in trials) and prominent barriers concerning cost and insurance access. Despite the inherent limitations of social media data, harnessing these unsolicited patient perspectives is crucial. It complements traditional research by identifying patient priorities, capturing real-world effectiveness and tolerability concerns, and underscoring the critical impact of accessibility. Understanding these patient-generated insights can inform clinical practice, guide support organizations, influence policy, and ultimately help bridge the gap between clinical trial evidence and the complexities of routine patient care for vitiligo.

## Abbreviations

FDA: Food and Drug Administration
JAK: Janus kinase
VADER: Valence Aware Dictionary and Sentiment Reasoner
